# Multi-epitope vaccines: a promising strategy against viral diseases in swine

**DOI:** 10.3389/fcimb.2024.1497580

**Published:** 2024-12-20

**Authors:** Xiaowei Chen, Yongfeng Li, Xiao Wang

**Affiliations:** ^1^ School of Basic Medical Sciences, Binzhou Medical University, Yantai, China; ^2^ Medicine and Pharmacy Research Center, Binzhou Medical University, Yantai, China; ^3^ State Key Laboratory for Animal Disease Control and Prevention, Harbin Veterinary Research Institute, Chinese Academy of Agricultural Sciences, Harbin, China

**Keywords:** viral diseases in swine, *in-silico* designment, multi-epitope vaccines, immunoinformatics, immunity

## Abstract

Viral infections in swine, such as African swine fever (ASF), porcine reproductive and respiratory syndrome (PRRS), and foot-and-mouth disease (FMD), have a significant impact on the swine industry. Despite the significant progress in the recent efforts to develop effective vaccines against viral diseases in swine, the search for new protective vaccination strategy remains a challenge. The antigenic epitope, acting as a fundamental unit, can initiate either a cellular or humoral immune response. Consequently, the combination of multi-epitopes expressing different stages of viral life cycle has become an optimal strategy for acquiring a potent, safe, and effective vaccine for preventing and treating viral diseases in swine. Recent progresses in immunoinformatic tools, coupled with an understanding of host immune responses and computational biology, have paved the way for innovative vaccine design disciplines that focus on computer-assisted, *in-silico* epitope prediction for the prevention of viral diseases in swine. The concept of multi-epitope vaccines driven by immunoinformatic methods has gained prominence in multiple studies, particularly in the development of vaccines targeting conserved epitopes in variable or rapidly mutating pathogens such as African swine fever virus (ASFV) and porcine reproductive and respiratory syndrome virus (PRRSV). In this review, we provide an overview of the *in-silico* design of the multi-epitope vaccines against viral diseases in swine, including the antigenicity, structural quality analysis, immune simulations, and molecular dynamics (MD) simulations. Furthermore, we also enumerate several multi-epitope vaccine applications that have shown promise to be against viral diseases in swine.

## 1 Introduction

Viral infections significantly impact the swine industry, particularly those affecting the respiratory, reproductive, and enteric systems. Examples of such diseases include African swine fever (ASF), porcine reproductive and respiratory syndrome (PRRS), classic swine fever (CSF), and foot-and-mouth disease (FMD). To counteract the viral diseases, swines are regularly vaccinated against these pathogens, which are responsible for considerable mortality, morbidity, and reduced weight gain. Traditional vaccines, including inactivated, live-attenuated, subunit, recombinant, and toxoid vaccines, have been instrumental in controlling and preventing viral diseases in swine. These vaccines have proven highly effective, safe, cost-efficient, and capable of inducing strong immune responses, thereby significantly reducing the burden of infectious diseases in swine populations (D’Amico et al., 2021). For instance, live vaccines, such as those used against rotavirus, transmissible gastroenteritis virus, pseudorabies virus, and parvovirus, have demonstrated notable efficacy. Particularly, some attenuated live vaccines confer long-lasting immunity by stimulating both antibody production and cellular immunity. The C-strain of the classical swine fever virus (CSFV), for example, is considered the gold standard for controlling CSF, providing swift and lifelong immunity after a single intramuscular inoculation (McCarthy et al., 2019; Li et al., 2024). However, the production of traditional vaccines often requires large-scale culturing of pathogens in controlled environments, a process that is complex, costly, and time-consuming (Vishweshwaraiah and Dokholyan, 2022; Poria et al., 2024). Moreover, live-attenuated vaccines, carry a small but inherent risk of the pathogen reverting to a virulent form and potentially causing disease, especially in immunocompromised individuals (Vishweshwaraiah and Dokholyan, 2022). Despite the availability of effective vaccines for some viral diseases in swine, the emergence of novel or mutant pathogens, as well as the potential for attenuated virus vaccines to revert or become latent, continue to pose significant economic challenges to the swine industry.

Advancements in bioinformatics tools and recombinant DNA technology have significantly facilitated the development of innovative strategies for designing and producing epitope-based vaccines. The application of immunoinformatic approaches in vaccine design has emerged as a promising strategy against viral diseases in swine. Notably, the concept of multi-epitope vaccines, designed using immunoinformatic methodologies, has gained considerable attention, especially for targeting conserved epitopes in variable or rapidly mutating pathogens. Antigenic epitopes are pivotal to be against viral diseases in swine, as they are the fundamental units that strongly elicit either cellular or humoral immune responses. Therefore, multi-epitope vaccines, which consist of a series of overlapping peptides, offers an effective and ideal approach for preventing and treating viral infections in swine. Compared to traditional vaccines and single-epitope vaccines, multi-epitope vaccines provide a comprehensive solution by incorporating cytotoxic T lymphocytes (CTLs), T helper (Th) and B cell epitopes, thereby simultaneously eliciting strong cellular and humoral immune responses simultaneously. Moreover, multi-epitope vaccines reduce the presence of undesirable components that may trigger pathological immune responses or adverse effects. These vaccines also broaden the spectrum of protection by encompassing multiple viral antigenic epitopes, thus targeting a wider range of viruses. Furthermore, the inclusion of adjuvant components enhances immunogenicity and prolongs immune responses. Well-designed multi-epitope vaccines with these characteristics hold significant promise as potent prophylactic and therapeutic agents against viral infections in swine. The integration of advanced bioinformatics and recombinant DNA technology in vaccine design not only accelerates the development process but also ensures the design of highly effective and safe vaccines tailored to the specific needs of the swine industry.

## 2 The process of the *in-silico* design of a multi-epitope vaccine against viral diseases in swine

**Figure 1:**
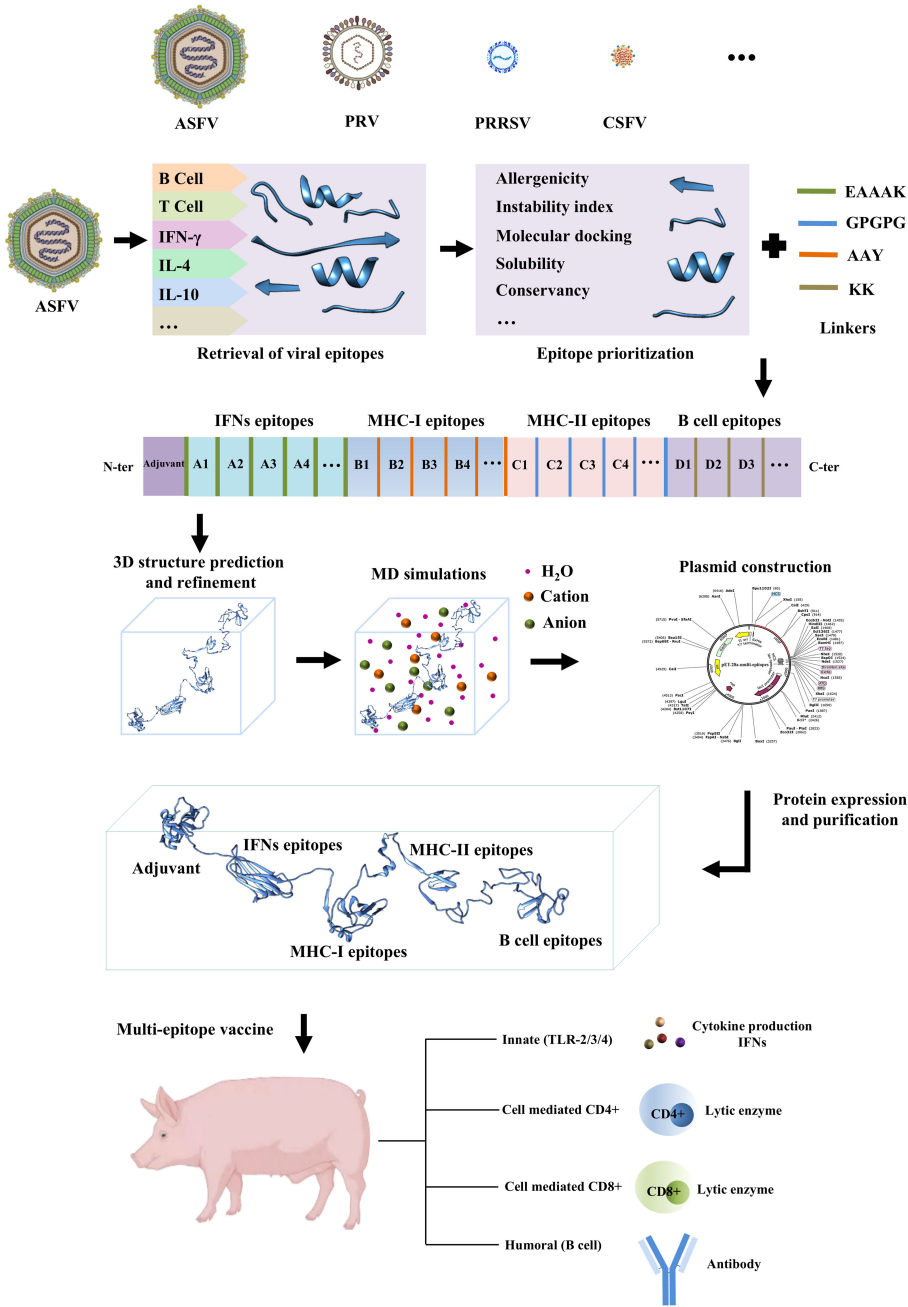
Methodological framework of the *in-silico* design of the multi-epitope vaccine and immune response. Multi-epitope vaccines can include a combination of cytotoxic T lymphocyte (CTL), T helper (Th), and B cell epitopes.

The *in-silico* design of a multi-epitope vaccine against viral diseases in swine typically involves a series of steps, as illustrated in **Figure 1** through the flowchart. Initially, viral protein sequences are retrieved from relevant databases. These sequences are then subjected to epitope prioritization utilizing various computational tools (**Table 1**). The predicted linear epitopes intended for the vaccine construct undergo further analysis to assess their allergenicity, molecular docking potential, degree of conservancy, and physicochemical properties. Following this assessment, the vaccine construct, which includes epitopes, linkers, and adjuvants, is modeled into a three-dimensional (3D) structure. This structure undergoes refinement and molecular dynamics (MD) simulations to ensure its stability and efficacy. Ultimately, the designed vaccine is analyzed using *in-silico* cloning techniques and subsequently evaluated experimentally to assess its ability to elicit immune protection.

**Table 1:** Webservers used in the multi-epitope prediction studies against viral diseases in swine.

Type		Tools	Webserver	References
T cell	MHC-I	EpiJen	http://www.ddg-pharmfac.net/epijen/EpiJen/EpiJen.htm	(Doytchinova et al., 2006)
		EPISOPT	http://bio.med.ucm.es/episopt.html	(Molero-Abraham et al., 2013)
		IEDB	http://tools.immuneepitope.org/mhci/	(Zhang et al., 2008)
		MAPPP	http://www.mpiib-berlin.mpg.de/MAPPP/	(Hakenberg et al., 2003)
		MHCPred	http://www.ddg-pharmfac.net/mhcpred/MHCPred/	(Guan et al., 2003)
		MULTIPRED2	http://cvc.dfci.harvard.edu/multipred2/index.php	(Zhang et al., 2011)
		NetCTL	http://www.cbs.dtu.dk/services/NetCTL/	(Larsen et al., 2005)
		NetMHC	http://www.cbs.dtu.dk/services/NetMHC/	(Nielsen et al., 2003)
		NetMHCpan 4.1	https://services.healthtech.dtu.dk/services/NetMHCpan-4.1/	(Reynisson et al., 2020)
		nHLApred	http://www.imtech.res.in/raghava/nhlapred/	(Bhasin and Raghava, 2007)
		PEPVAC	http://imed.med.ucm.es/PEPVAC/	(Reche and Reinherz, 2005)
		Propred-1	http://www.imtech.res.in/raghava/propred1/	(Singh and Raghava, 2003)
		Rankpep	http://imed.med.ucm.es/Tools/rankpep.html	(Reche and Reinherz, 2007)
		SVMHC	http://abi.inf.uni-tuebingen.de/Services/SVMHC/	(Dönnes and Elofsson, 2002)
		SVRMHC	http://us.accurascience.com/SVRMHCdb/	(Liu et al., 2006)
	MHC-II	EpiDOCK	http://epidock.ddg-pharmfac.net	(Atanasova et al., 2013)
		EpiTOP	http://www.pharmfac.net/EpiTOP	(Dimitrov et al., 2010)
		IEDB-MHCII	http://tools.immuneepitope.org/mhcii/	(Zhang et al., 2008)
		MHC2PRED	http://www.imtech.res.in/raghava/mhc2pred/index.html	(Bhasin and Raghava, 2004)
		MHCPred	http://www.ddg-pharmfac.net/mhcpred/MHCPred/	(Guan et al., 2003)
		MULTIPRED2	http://cvc.dfci.harvard.edu/multipred2/index.php	(Zhang et al., 2011)
		NetMHCII	http://www.cbs.dtu.dk/services/NetMHCII/	(Nielsen et al., 2007)
		NetMHCIIpan	http://www.cbs.dtu.dk/services/NetMHCIIpan/	(Nielsen et al., 2008)
		PREDIVAC	http://predivac.biosci.uq.edu.au/	(Oyarzún et al., 2013)
B cell		ABCpred	http://www.imtech.res.in/raghava/abcpred/	(Saha and Raghava, 2006b)
		BCPREDS	http://ailab.ist.psu.edu/bcpred/	(El-Manzalawy et al., 2008)
		BepiPred 3.0	https://services.healthtech.dtu.dk/services/BepiPred-3.0/	(Clifford et al., 2022)
		CBTOPE	http://www.imtech.res.in/raghava/cbtope/submit.php	(Ansari and Raghava, 2010)
		CEP	http://bioinfo.ernet.in/cep.htm	(Kulkarni-Kale et al., 2005)
		DiscoTope-3.0	https://services.healthtech.dtu.dk/services/DiscoTope-3.0/	(Høie et al., 2024)
		ElliPro	http://tools.iedb.org/ellipro/	(Ponomarenko et al., 2008)
		EPCES	http://sysbio.unl.edu/services/EPCES/	(Liang et al., 2009)
		EPIPRED	http://opig.stats.ox.ac.uk/webapps/sabdab-sabpred/EpiPred.php	(Krawczyk et al., 2014)
		EpiSearch	http://curie.utmb.edu/episearch.html	(Negi and Braun, 2009)
		EPITOPIA	http://epitopia.tau.ac.il/	(Rubinstein et al., 2009)
		LBtope	http://www.imtech.res.in/raghava/lbtope/	(Singh et al., 2013)
		SVMtrip	http://sysbio.unl.edu/SVMTriP/prediction.php	(Yao et al., 2012)
		VaxiJen v3.0	https://www.ddg-pharmfac.net/vaxijen3/home/	(Doneva and Dimitrov, 2024)
IFN-γ		IFNepitope	http://crdd.osdd.net/raghava/ifnepitope/	(Dhanda et al., 2013b)
IL-4		IL4pred	http://webs.iiitd.edu.in/raghava/il4pred	(Dhanda et al., 2013a)
IL-10		IL10pred	http://webs.iiitd.edu.in/raghava/il10pred	(Nagpal et al., 2017)
Allergenicity		AlgPred	https://webs.iiitd.edu.in/raghava/algpred/submission.html	(Saha and Raghava, 2006a)
		AllerCatPro 2.0	https://allercatpro.bii.a-star.edu.sg/	(Nguyen et al., 2022)
		ALLERDET	http://allerdet.frangam.com/	(Garcia-Moreno and Gutiérrez-Naranjo, 2022)
		AllerTOP v.2.0	https://www.ddg-pharmfac.net/AllerTOP/	(Dimitrov et al., 2014)
		iAller	https://github.com/laihongyan/iAller	(Liu et al., 2023)
		Allermatch	https://allermatch.org/allermatchsearch/form	(Fiers et al., 2004)
		ProAll-D	https://data.mendeley.com/datasets/tjmt97xpjf/1	(Shanthappa and Kumar, 2022)
Conservancy		IEDB	http://tools.iedb.org/conservancy/	(Bui et al., 2007)

### 2.1 Retrieving viral protein sequences and sequence alignment

Viral protein sequences are typically retrieved from databases such as the National Center for Biotechnology Information (NCBI), UniProt, or other similar resources. Following retrieval, multiple sequence alignment of the viral proteins is performed to generate consensus sequences. These consensus sequences are subsequently analyzed to assess their conservation relative to reference sequences. Mutant amino acid sites are identified and excluded from further analysis. Additionally, signal peptide regions are removed from the candidate antigen proteins to ensure the focus remains on the functional regions of the protein relevant for vaccine development.

### 2.2 Epitope mapping and selection of the epitope segments

**Figure 2:**
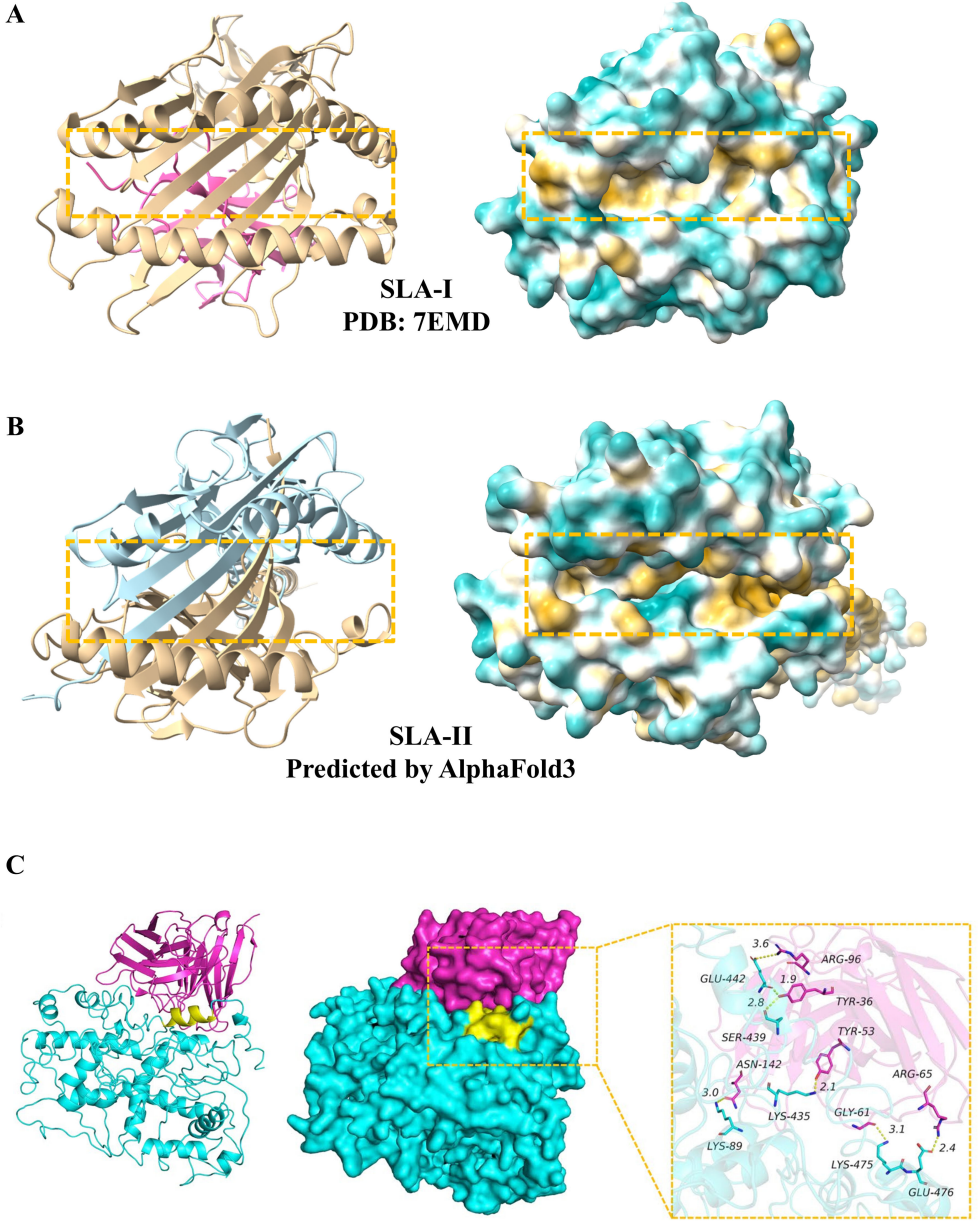
Structures of the SLA-I, SLA-II and antibody molecules. **(A)** Ribbon and surface representations of the SLA-I molecule (PDB: 7EMD). **(B)** Ribbon and surface representations of the SLA-II molecule predicted by AlphaFold3. **(C)** Ribbon and surface representations of the localization of the mAb 7E7 epitope in ASFV pB602L modified based on Song et al., 2024. The binding of mAb 7E7 to ASFV pB602L was shown. Yellow: epitope amino acid residues; Red, mAb 7E7; Cyan, ASFV pB602L.

A comprehensive prediction of antigenic, non-toxic, and homologous epitopes is essential to induce T cell, B cell responses, and IFN-γ. The major histocompatibility complex (MHC) class I (MHC-I) and class II (MHC-II) are critical for T cell-mediated immune responses to viral infections, as they present viral protein fragments to CD8+ and CD4+ T cells, respectively. In swine, this system is known as the swine leukocyte antigen (SLA), encompassing SLA-I (**Figure 2A**) and SLA-II (**Figure 2B**), while in humans, it is referred to as the human leukocyte antigen (HLA) system. Based on the analysis of retrieved viral protein sequences, T-cell epitopes were predicted using two primary MHC-I and MHC-II binding tools (**Table 1**). Notably, SLA-II molecular information is not yet reported. However, SLA-II shares similarities with human MHC-II in sequence and structure, as determined through sequential alignment and homology modeling. Here, the structure of SLA-II was predicted by AlphaFold3 (**Figures 2B** and **S1**), indicating a similar structure with human MHC-II structure. Consequently, SLA-II epitopes were predicted using human MHC-II online server tools (**Table 1**).

B cells, which differentiate into plasmocytes to produce antibodies, are crucial for humoral immunity. The binding mode of the epitope with its antibody was presented (**Figure 2C**). B cell epitopes can be predicted using the tools listed in **Table 1**. Alongside the high binding affinity to MHC-II molecules and significant recognition potential by T-cell receptors, some final helper T-cell (HTL) epitopes also exhibit cytokine-inducing capabilities, specifically for IFN-γ, IL-4, and IL-10. IFN-γ regulates viral replication by stimulating natural killer cells and macrophages to combat viral infections in swine, whereas IL-4 and IL-10, as anti-inflammatory cytokines, contribute to immunoregulatory responses, thus supporting a balanced and safe vaccine profile. Finally, various epitope prediction tools (**Table 1**) have been utilized to identify potential epitopes for viral proteins. All predicted epitopes have been aggregated, with high-ranking regions overlapping, to construct a multi-epitope vaccine capable of eliciting cellular T-cell, helper T-cell, B-cell, IFN-γ, IL-4, and IL-10 responses.

### 2.3 Evaluation of the multi−epitope vaccine on allergenicity, conservancy, physicochemical properties

The allergenicity and conservancy of the predicted epitopes have to be further considered to ensure their suitability for mapping in the design of a multi-epitope vaccine. Several tools can be used to assess allergenicity and conservancy of the predicted epitopes (**Table 1**). Additionally, the physicochemical properties of the epitopes, which primarily include amino acid composition, molecular weight (MW), instability index, solubility index, *in vitro* and *in vivo* half-life, grand average of hydropathicity (GRAVY), and theoretical isoelectric point (pI), should also be considered.

### 2.4 Construction of multi−epitope vaccine, structure prediction, refinement, and quality analysis

Due to the small size of individual epitope peptides, each epitope alone typically cannot elicit a sufficient immune response when used in a vaccine. To enhance the immunogenicity and achieve higher antibody concentrations compared to a single immunogen, the use of linkers is essential in vaccine design. Consequently, the final multi-epitope vaccine was constructed by sequentially joining the CTL, HTL, and B cell epitopes using appropriate linkers. The EAAAK linker was used to connect the N-terminal of the vaccine to the cholera enterotoxin subunit B (CTB) adjuvant. This linker, which forms a salt bridge between lysine and glutamic acid, leads to the creation of a helix structure. This helix stabilizes the protein’s structure and function, enhancing its overall efficacy (Chao et al., 2024). In contrast, other linkers such as AAY, GPGPG, and KK were utilized to link cytotoxic T lymphocyte (CTL), helper T lymphocyte (HTL), and B cell epitopes, respectively. The AAY linker, which serves as a cleavage site for proteasomes in mammalian cells, reduces the junctional immunogenicity of multi-epitopes. This reduction in immunogenicity helps in optimizing the presentation of multiple epitopes without overwhelming the immune system (Yang et al., 2015). The GPGPG linker is crucial for generating HTL responses, which are essential for the efficacy of multi-epitope vaccines. Additionally, it reduces junctional immunogenicity and stabilizes the immunogenicity of specific epitopes, enhancing the overall effectiveness of the vaccine (Livingston et al., 2002). The KK linker, targeted by the lysosomal protease Cathepsin B, aids in the presentation of antigen restricted by MHC-II molecules. This process is critical for the effective presentation of antigens to the immune system, ensuring a robust immune response (Aram et al., 2024). Furthermore, the addition of adjuvants to subunit vaccines is recommended to enhance immune responses and improve vaccine efficacy. Adjuvants can stimulate the immune system, making it more responsive to the vaccine antigens and thereby improving the overall effectiveness of the vaccine (Orosco and Espiritu, 2024). These linkers and adjuvants play critical roles in the design and development of multi-epitope vaccines, ensuring that they effectively stimulate the immune system and provide robust protection against viral diseases in swine.

For structural prediction, the secondary structure of the multi-epitope vaccine was assessed using PDBsum and SOPMA online analysis software. The tertiary structures were predicted with several tools, including 3Dpro, Robetta, Rosetta, AlphaFold2, and AlphaFold3. To enhance the quality of the structure and improve protein stability, the initial 3D model of the vaccine was refined using the GalaxyRefine server (https://galaxy.seoklab.org/cgi-bin/submit.cgi?type=REFINE). The refined tertiary structure of the final multi-epitope vaccine protein was validated through structure validation servers, SAVES v6.0 (https://saves.mbi.ucla.edu/) and ProSA (https://prosa.services.came.sbg.ac.at/prosa.php). SAVES v6.0 comprises a comprehensive suite of five programs that assess the overall consistency of protein structures. Among these, VERIFY-3D is commonly employed to evaluate the 3D sequence profile for protein models, while PROCHECK is utilized to validate the structure using Ramachandran plots, which display the favorable regions of backbone dihedral angles in relation to amino acid residues. ProSA calculates a z-score to indicate the overall quality of the model and measures the deviation of the total energy of the structure against an energy distribution derived from random conformations. Additionally, ProSA provides an energy plot that illustrates local model quality by plotting energies as a function of amino acid sequence position.

### 2.5 Molecular docking of the multi−epitope with toll like receptors

Molecular docking is crucial in computational vaccine design as it elucidates how a vaccine interacts with immune receptors. By simulating the docking process, it is possible to predict the binding affinity and stability of the vaccine-receptor complex, which is crucial for selecting and optimizing vaccine candidates. This process ultimately contributes to the design of more effective and targeted vaccines. For instance, the interaction of the multi-epitope vaccine with Toll-like receptors (TLRs) can enhance the vaccine’s efficacy (Vijay, 2018). Protein-protein docking was employed to assess interactions between the multi-epitope vaccine and TLR proteins, including TLR3, TLR4, and TLR8. The 3D structures of TLR3 (PDB ID: 1ZIW), TLR4 (PDB ID: 4G8A), and TLR8 (PDB ID: 3W3G) were retrieved from the Protein Data Bank and used as receptors in the docking procedure. Various popular docking tools were utilized, including ClusPro, PatchDock, HawkDock and HADDOCK. The best docking results were selected based on the free binding energy of the ligand-receptor complexes. By docking the vaccine with TLRs, researchers can evaluate how the multi-epitope vaccine stimulates the immune system and potentially enhances the immune response against viral infections in swine.

### 2.6 Immune simulation of the multi−epitope vaccine

The host immune response to the multi-epitope vaccine construct was evaluated using the C-ImmSimm server (https://kraken.iac.rm.cnr.it/C-IMMSIM/) (Rapin et al., 2010; Ragone et al., 2021). This analysis predicts the induction of adaptive immunity through the use of position-specific scoring matrix (PSSM) and machine learning techniques for epitope and immune interaction predictions. C-ImmSimm employs a three-dimensional (3D) stochastic cellular automaton model, which represents major cell types from both the lymphoid and myeloid lineages. The lymphoid lineage includes T helper lymphocytes (Th), cytotoxic T lymphocytes (CTL), B lymphocytes, and antibody-producing plasma cells (PLB). The myeloid lineage includes macrophages (M) and dendritic cells (DC). These cellular entities interact according to a set of predefined rules that simulate the various phases of the immune system’s recognition and response processes against a pathogen.

### 2.7 Molecular dynamics simulations

MD simulation is a powerful computational technique employed to study the movement and behavior of atoms and molecules over time. It is essential for understanding the dynamics and interactions of biological molecules such as proteins and small molecules. By simulating their motions, MD provides valuable insights into structural changes, binding events, and functional mechanisms of these molecules. This makes it an essential tool for evaluating the quality of multi-epitope constructs, particularly in the context of vaccine design. To evaluate the quality of multi-epitope constructs, various parameters are analyzed, including root mean square deviation (RMSD), root mean square fluctuation (RMSF), radius of gyration (Rg), and intramolecular hydrogen bonds. These analyses are conducted using molecular dynamics simulation tools such as GROMACS, LAMMPS, and NAMD. The MD simulation process consists of three main steps: system preparation, pre-processing, and simulation. Initially, a force field is used to generate the topology for the protein-ligand complex in all MD simulations. The multi-epitope constructs are then solvated in a cubic water box to mimic the aqueous environment of biological systems. Ions are introduced using a genion tool to neutralize the charged protein complex. To avoid steric clashes and ensure proper geometry, the solvated system undergoes energy minimization. Subsequently, system equilibration is performed through NVT (number of particles (N), volume (V), and temperature (T)) and NPT (number of particles (N), pressure, and temperature (T)) ensembles without restraints. Finally, MD simulations are carried out to analyze the trajectory of the multi-epitope constructs. By employing MD simulations, we can obtain detailed insights into the behavior and stability of multi-epitope constructs, which is crucial for the design and optimization of effective vaccines. The integration of MD simulation tools with bioinformatics and recombinant DNA technology further enhances the precision and efficiency of multi-epitope vaccine development, making it a potent approach for combating viral infections in swine.

### 2.8 *In-silico* codon optimization and cloning

For cloning, codon optimization was usually performed by the online tools such as Java Codon Adaptation Tool (JCAT), avoiding rho-independent transcription terminators and prokaryotic ribosome binding sites. Usually, *E.coli* (Strain K12) was used to express the vaccine protein. Codon adaptation index (CAI), the guanine and cytosine (GC) contents and Codon Frequency and Distribution (CFD) were evaluated by GenScript server. To enhance the expression efficiency of the final vaccine protein, pET28a (+) was selected as the vector to express the vaccine protein. The restriction cloning module of the SnapGene tool (http://www.snapgene.com) was employed to clone the adapted nucleotide sequence into the pET-28a (+) vector with suitable restriction enzymes added at the N-terminal and C-terminal sites, respectively. By codon optimization and cloning, we can efficiently clone and express optimized vaccine proteins in *E. coli*, ensuring high yields and purity of the target protein for further use in multi-epitope vaccine development.

## 3 The application of the multi-epitope vaccines against viral diseases in swine

Many multi-epitope vaccines targeting viral diseases in swine have been reported in recent years. Here, we reviewed and summarized several epitope-based vaccines for key porcine viruses, including ASFV, PRRSV, and FMDV (**Supplementary Table S1**), which impact the respiratory, reproductive, and enteric systems of pigs. Additionally, we have included epitopes from other relevant porcine viruses, such as classical swine fever virus (CSFV) and Pseudorabies virus (PRV) (**Supplementary Table S1**). Notably, the identification of T cell epitopes in PRV remains relatively underexplored, based on our search of PubMed and Google Scholar. We acknowledge that due to the extensive body of research on multi-epitope vaccines for viral diseases in swine, we were unable to compile a comprehensive list of all reported epitopes and corresponding references.

ASF poses a significant challenge to the global swine industry due to its 100% mortality rate, and there is currently no effective vaccine available (Urbano and Ferreira, 2022). The challenges in developing ASFV vaccines are largely attributed to the virus’s intricate nature, characterized by a genome containing 150 to 167 genes (Blome et al., 2020). Consequently, identifying essential elements to induce a robust and protective immune response is difficult because many of the viral genes have unknown functions. Moreover, the high genetic variability among ASFV strains further complicates the development of a universal vaccine that can provide broad protection against diverse viral variants. The design of multi-epitope vaccines against ASFV has increasingly been guided by immunoinformatics approaches. These multi-epitope vaccines are designed to elicit both T-cell and B-cell immunogenic responses. One such vaccine, based on the ASFV p72 protein, has demonstrated high sensitivity (85.7%) and specificity (97.6%) (Zhang et al., 2021b). Additionally, several linear epitope vaccines have been identified to elicit both T-cell and B-cell immunogenic responses (**Supplementary Table S1**). For example, the SKENLTPDE epitope located in the C-terminus of ASFV pB602L has been identified as a novel conserved B-cell epitope, specifically binding to monoclonal antibody 7E7 (Song et al., 2024). Similarly, the IADAINQEF epitope from ASFV p34 has been recognized as a T-cell epitope. Furthermore, four linear B-cell epitopes-HKPHQSKPIL, PVGFEYENKV, VNGNSLDEYSS, and GYKHLVGQEV-derived from ASFV p72, have been recognized by monoclonal antibodies (Miao et al., 2023). These developments highlight the potential of multi-epitope and linear epitope vaccines as promising strategies for combating African swine fever.

PRRSV is a significant pathogen that causes severe reproductive failure in sows and respiratory distress and mortality in young pigs, resulting in substantial economic losses for the global pig industry. Two multi-epitope subunit vaccines based on the conserved B-cell epitopes of the viral proteins Cp1 and Cp2 have been constructed and tested in both mice and piglets, demonstrating high levels of neutralizing antibodies (Chen et al., 2012). Additionally, the combination of Cp1 and Cp2 with the N-terminal 22-370 amino acids (aa) of porcine gp96 (Gp96N) as an adjuvant was shown to induce 3-4-fold higher titers of Cp1 and Cp2 neutralizing antibodies. In addition to these efforts, a bivalent multi-epitope vaccine targeting both PRRSV and Mycoplasma hyopneumoniae (Mhp) has been reported to elicit robust humoral and cellular immune responses in mice (Gao et al., 2022). Furthermore, a recombinant swinepox virus expressing a multi-epitope peptide from a PRRSV mimotope P2 has been shown to neutralize monoclonal antibodies and provide significant protection against PRRSV infection in pigs (Lin et al., 2017).

FMDV remains an important pathogen affecting livestock. Numerous multi-epitope vaccines targeting FMDV have been developed and reported. One such DNA vaccine, encoding FMDV B- and T-cell multi-epitopes and targeting class II swine leukocyte antigens, has demonstrated protective effects in pigs challenged with FMDV. This protection is achieved through the induction of specific interferon-gamma (IFNγ)-secreting T-cells and the rapid generation of neutralizing antibodies (Borrego et al., 2011). Efforts to enhance the efficacy of the FMDV multi-epitope vaccine have led to the utilization of mannan-decorated inulin acetate microparticles (M-IA MPs). These microparticles have significantly boosted levels of antigen-specific immunoglobulin G (IgG), IgG1, IgG2a, and anti-FMDV antibodies (Yoon et al., 2019). Similarly, the fusion of heparin-binding hemagglutinin (HBHA)—a novel Toll-like receptor 4 (TLR4) agonist—with a multi-epitope immunogen derived from FMDV serotypes A and O has shown enhanced efficacy in activating murine dendritic cells via the TLR4 pathway. Furthermore, the incorporation of HBHA was found to elevate cellular immune responses by inducing intracellular cytokine expression in both Th1 and Th2 cells, including IFN-γ, tumor necrosis factor-alpha (TNF-α), interleukin-4 (IL-4), IL-6, and IL-10 (Lei et al., 2020). Recently, a recombinant FMDV multiple-epitope trivalent vaccine based on three distinct topotypes has undergone evaluation for its immune efficacy in pigs. The results showed complete protection against challenges from the three corresponding virus strains (Shao et al., 2024). Additionally, the coupling of M cell-targeting ligand Co1 to multi-epitope TB1 of FMDV has been identified to improve the delivery efficiency of the multi-epitope protein antigen TB1. This coupling effectively induces mucosal secretory IgA (SIgA) and IgG secretion, promotes robust cell-mediated immune responses, enhances T lymphocyte proliferation in the spleen, and elevates intracellular cytokine levels, including IL-2, IFN-γ, IL-10, and IL-5 (Zhang et al., 2021a).

## 4 Discussion

Currently, the majority of multi-epitope vaccines for viral diseases in swine are still at the *in-silico* design stage, with limited application. These vaccines have primarily been validated through computational immune simulations rather than experimental studies. In contrast, many linear epitopes have been experimentally validated for their efficacy against viral infections in swine, employing both computational and experimental approaches. As the experimental validation of linear epitopes advances, the construction and assembly of multi-epitope vaccines will become more feasible. Nonetheless, multi-epitope vaccines still hold significant promise for combating viral diseases in swine, potentially advancing vaccine development. Several multi-epitope vaccines are already undergoing pre-clinical and clinical trials. For example, a multi-epitope vaccine targeting the T-cell receptor alternate reading frame protein (TARP) is currently in pre-clinical trials (Oh et al., 2004; Epel et al., 2008; Wood et al., 2016). The vaccine includes two synthetic 9-mer TARP peptides and five additional 20-mer peptides that overlap by 10-mer and cover the entire 58-residue sequence of the TARP. The overlapping nature of these additional peptides enables them to stimulate both humoral and cellular killing responses, leading to the treatment of prostate and breast cancer. Another example is a multi-epitope vaccine containing 12 MHC-I restricted melanoma peptides, a tetanus peptide, and six melanoma helper peptides, which has progressed to a randomized phase II trial against metastatic melanoma (Slingluff et al., 2013). Furthermore, a multi-peptide cancer vaccine with granulocyte-macrophage colony-stimulating factor (GM-CSF) is currently undergoing a phase I/II study in patients with advanced melanoma (Perales et al., 2008). These findings indicate that multi-epitope vaccines present significant promise for the prevention and treatment of viral diseases in swine.

## 5 Conclusion

Immunoinformatics methods play a crucial role in predicting and screening suitable epitopes for the design of effective multi-epitope vaccines. When combined with experimental validation, multi-epitope vaccines emerge as a promising strategy for preventing and treating viral infections in swine.

## Supplementary material

Supplementary Figure 1 The confidence metrics of the predicted structure of SLA-II by pLDDT, PAE predicted aligned error (PAE), and pTM and ipTM scores.
