# Supplementary figures and images for: Multi-epitope vaccines: a promising strategy against viral diseases in swine
Source: Front Cell Infect Microbiol. 2024 Dec 20;14:1497580. doi: 10.3389/fcimb.2024.1497580 (PMC11695243; doi:10.3389/fcimb.2024.1497580)

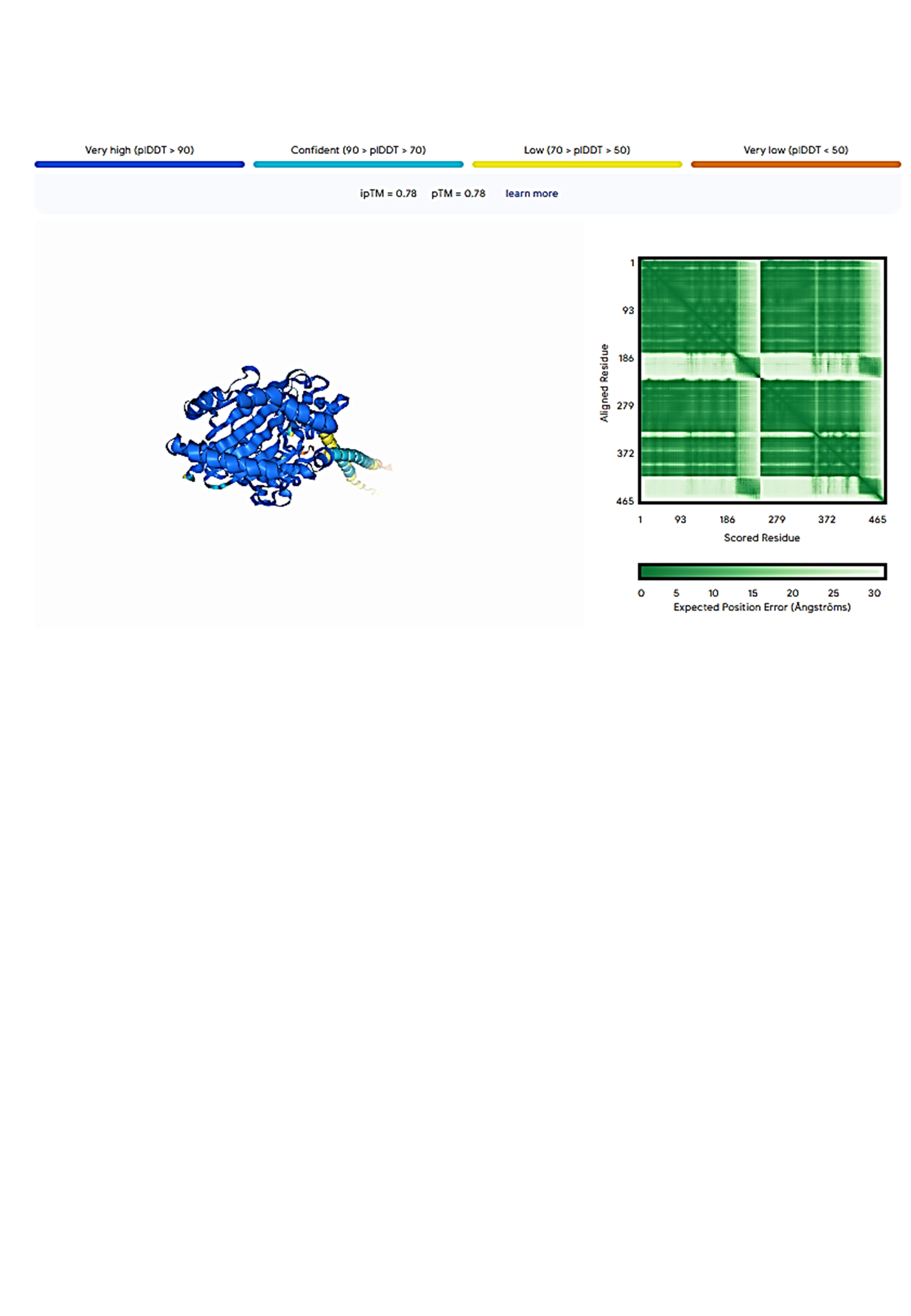

Supplement: Supplementary Figure 1 — The confidence metrics of the predicted structure of SLA-II by pLDDT, PAE predicted aligned error (PAE), and pTM and ipTM scores. [file Image1.tif]
