# Supplementary material for: Multi-epitope vaccines: a promising strategy against viral diseases in swine
Source: Front Cell Infect Microbiol. 2024 Dec 20;14:1497580. doi: 10.3389/fcimb.2024.1497580 (PMC11695243; doi:10.3389/fcimb.2024.1497580)
Supplement: Supplementary file 2 [file DataSheet1.docx]

**Table S1 The identified T and B cell epitopes of several porcine viruses for the construction of a multi-epitope vaccine.**

| Porcine viruses | Epitopes | | References |
| --- | --- | --- | --- |
| ASFV | T cell | AAAIEEEDI | (Yue et al., 2022) |
|  |  | YGDFFHDMV |  |
|  |  | SLDEYSSDV |  |
|  |  | IADAINQEF | (Zhang et al., 2023) |
|  |  | TTKTLLSEL | (Song et al., 2023) |
|  | B cell | SKENLTPDE | (Song et al., 2024) |
|  |  | VNGNSLDEYSS | (Miao et al., 2023) |
|  |  | GYKHLVGQEV |  |
|  |  | PVGFEYENKV |  |
|  |  | HKPHQSKPIL |  |
| PRRSV | T cell | LAALICFVIRLAKNC | (Gao et al., 2022) |
|  |  | RLYRWRSPV |  |
|  |  | CNDSTAPQKVLLAFS |  |
|  |  | FGYMTFVHFESTNRV |  |
|  |  | DLSPEGENAVYV |  |
|  | B cell | Cp1 and Cp2 based on the viral proteins with the N-ter 22-370 residues of porcine gp96 | (Chen et al., 2012)  (Lin et al., 2017) |
|  |  | mimotope P2 |  |
|  |  | FVVRRPGSTTVNGTLVPGLKSLVLGGR | (Gao et al., 2022) |
|  |  | AVKQGVVNLVKYAK |  |
|  |  | SSHIQLIYNLTLCELNG |  |
| FMDV | T cell | ATEIRELLV | (Yue et al., 2022) |
|  |  | Residues 20-34 of the VP4 | (Borrego et al., 2011) |
|  |  | Residues 21-35 of the 3A | (Blanco et al., 2001; Lee et al., 2017) |
|  |  | LRTATYYFADLEVAV | (Guzman et al., 2010) |
|  |  | Residues 113-132 of the VP2 | (Li et al., 2023) |
|  | B cell | Residues 133-156 of the VP1 | (Borrego et al., 2011)  (Borrego et al., 2011) |
|  |  | residues 11-40 of the 3C |  |
|  |  | residues 136-162 of the VP1 | (Lee et al., 2017) |
|  |  | KYDESPVTNVRGDLQVLAQKAARTLP | (Shao et al., 2024)  (Shao et al., 2024) |
|  |  | RHKQKIVAPVKQLL |  |
| CSFV | T cell | STEEMGDDF | (Zhao et al., 2023) |
|  |  | ATDRHSDYF | (Zhao et al., 2023) |
|  |  | PESRKKLEKALLAWA | (Franzoni et al., 2013) |
|  |  | YEPRDSYF | (Franzoni et al., 2013) |
|  |  | KHKVRNEVMVHWFDD | (Armengol et al., 2002) |
|  | B cell | RYLASLHKKALPT | (Chang et al., 2012) |
|  |  | YRYAIS | (Xu et al., 2021) |
|  |  | HECLIG |  |
|  |  | RX(D/E)K(R)XFXXR | (Mi et al., 2024) |
|  |  | LHXGXLLT |  |
| PRV | T cell | No report |  |
|  | B cell | RRGRFRSPDAD | (Zhang et al., 2019) |
|  |  | SAVATAA |  |
|  |  | RLRRE | (Xu et al., 2019) |
|  |  | EMGIGDY |  |
|  |  | SGGVLDALK | (Du et al., 2016) |

**References**

Armengol, E., Wiesmüller, K.-H., Wienhold, D., Büttner, M., Pfaff, E., Jung, G., et al. (2002). Identification of T-cell epitopes in the structural and non-structural proteins of classical swine fever virus. *Journal of General Virology* 83, 551–560. doi: 10.1099/0022-1317-83-3-551

Blanco, E., Garcia-Briones, M., Sanz-Parra, A., Gomes, P., Oliveira, E. D., Valero, M. L., et al. (2001). Identification of T-Cell Epitopes in Nonstructural Proteins of Foot-and-Mouth Disease Virus. *Journal of Virology*. 75, 3164-3174. doi: 10.1128/jvi.75.7.3164-3174.2001

Borrego, B., Argilaguet, J. M., Pérez-Martín, E., Dominguez, J., Pérez-Filgueira, M., Escribano, J. M., et al. (2011). A DNA vaccine encoding foot-and-mouth disease virus B and T-cell epitopes targeted to class II swine leukocyte antigens protects pigs against viral challenge. *Antiviral Res* 92, 359–363. doi: 10.1016/j.antiviral.2011.07.017

Chang, C.-Y., Huang, C.-C., Deng, M.-C., Huang, Y.-L., Lin, Y.-J., Liu, H.-M., et al. (2012). Antigenic mimicking with cysteine-based cyclized peptides reveals a previously unknown antigenic determinant on E2 glycoprotein of classical swine fever virus. *Virus Res* 163, 190–196. doi: 10.1016/j.virusres.2011.09.019

Chen, C., Li, J., Bi, Y., Jia, X., Meng, S., Sun, L., et al. (2012). Gp96 enhances the immunogenicity of subunit vaccine of porcine reproductive and respiratory syndrome virus. *Virus Research* 167, 162–172. doi: 10.1016/j.virusres.2012.04.011

Du, W., Wang, Y., Huang, L., Wei, Y., Chen, D., Sun, J., et al. (2016). Characterization of monoclonal antibodies that recognize the amino- and carboxy-terminal epitopes of the pseudorabies virus UL42 protein. *Appl Microbiol Biotechnol* 100, 181–192. doi: 10.1007/s00253-015-6957-7

Franzoni, G., Kurkure, N. V., Essler, S. E., Pedrera, M., Everett, H. E., Bodman-Smith, K. B., et al. (2013). Proteome-wide screening reveals immunodominance in the CD8 T cell response against classical swine fever virus with antigen-specificity dependent on MHC class I haplotype expression. *PLoS One* 8, e84246. doi: 10.1371/journal.pone.0084246

Gao, Z., Chen, L., Song, T., Pan, X., Li, X., Lu, G., et al. (2022). A candidate multi-epitope vaccine against porcine reproductive and respiratory syndrome virus and Mycoplasma hyopneumoniae induces robust humoral and cellular response in mice. *Vaccine* 40, 2370–2378. doi: 10.1016/j.vaccine.2022.03.021

Guzman, E., Taylor, G., Charleston, B., and Ellis, S. A. (2010). Induction of a cross-reactive CD8(+) T cell response following foot-and-mouth disease virus vaccination. *J Virol* 84, 12375–12384. doi: 10.1128/JVI.01545-10

Lee, H.-B., Piao, D.-C., Lee, J.-Y., Choi, J.-Y., Bok, J.-D., Cho, C.-S., et al. (2017). Artificially designed recombinant protein composed of multiple epitopes of foot-and-mouth disease virus as a vaccine candidate. *Microb Cell Fact* 16, 33. doi: 10.1186/s12934-017-0648-2

Li, Q., Wubshet, A. K., Wang, Y., Heath, L., and Zhang, J. (2023). B and T Cell Epitopes of the Incursionary Foot-and-Mouth Disease Virus Serotype SAT2 for Vaccine Development. *Viruses* 15, 797. doi: 10.3390/v15030797

Lin, H., Ma, Z., Hou, X., Chen, L., and Fan, H. (2017). Construction and immunogenicity of a recombinant swinepox virus expressing a multi-epitope peptide for porcine reproductive and respiratory syndrome virus. *Sci Rep* 7, 43990. doi: 10.1038/srep43990

Mi, S., Bao, F., Liu, Z., Zhang, Y., Li, H., Wu, M., et al. (2024). Generation and epitope mapping of novel neutralizing monoclonal antibodies against glycoprotein E2 of CSFV. *International Journal of Biological Macromolecules* 282, 136609. doi: 10.1016/j.ijbiomac.2024.136609

Miao, C., Yang, S., Shao, J., Zhou, G., Ma, Y., Wen, S., et al. (2023). Identification of p72 epitopes of African swine fever virus and preliminary application. *Front. Microbiol.* 14, 1126794. doi: 10.3389/fmicb.2023.1126794

Shao, J., Liu, W., Gao, S., Chang, H., and Guo, H. (2024). A recombinant multi-epitope trivalent vaccine for foot-and-mouth disease virus serotype O in pigs. *Virology* 596, 110103. doi: 10.1016/j.virol.2024.110103

Song, J., Wang, M., Zhou, L., Tian, P., Sun, J., Sun, Z., et al. (2024). A novel conserved B-cell epitope in pB602L of African swine fever virus. *Appl Microbiol Biotechnol* 108, 78. doi: 10.1007/s00253-023-12921-6

Song, J., Wang, M., Zhou, L., Tian, P., Sun, Z., Sun, J., et al. (2023). A candidate nanoparticle vaccine comprised of multiple epitopes of the African swine fever virus elicits a robust immune response. *Journal of Nanobiotechnology* 21, 424. doi: 10.1186/s12951-023-02210-9

Xu, H., Han, G., Lu, Y., Liu, Z., Tao, L., and He, F. (2021). Broad neutralization of CSFV with novel monoclonal antibodies in vivo. *Int J Biol Macromol* 173, 513–523. doi: 10.1016/j.ijbiomac.2021.01.142

Xu, J., Wu, J., Cheng, X., Tong, W., Zheng, H., Zhu, H., et al. (2019). Identification of two novel epitopes targeting glycoprotein E of pseudorabies virus using monoclonal antibodies. *Biochemical and Biophysical Research Communications* 519, 330–336. doi: 10.1016/j.bbrc.2019.08.168

Yue, C., Xiang, W., Huang, X., Sun, Y., Xiao, J., Liu, K., et al. (2022). Mooring Stone-Like Arg114 Pulls Diverse Bulged Peptides: First Insight into African Swine Fever Virus-Derived T Cell Epitopes Presented by Swine Major Histocompatibility Complex Class I. *J Virol* 96, e0137821. doi: 10.1128/JVI.01378-21

Zhang, P., Lv, L., Sun, H., Li, S., Fan, H., Wang, X., et al. (2019). Identification of linear B cell epitope on gB, gC, and gE proteins of porcine pseudorabies virus using monoclonal antibodies. *Veterinary Microbiology* 234, 83–91. doi: 10.1016/j.vetmic.2019.05.013

Zhang, X., Guan, X., Wang, Q., Wang, X., Yang, X., Li, S., et al. (2023). Identification of the p34 Protein of African Swine Fever Virus as a Novel Viral Antigen with Protection Potential. *Viruses* 16, 38. doi: 10.3390/v16010038

Zhao, X., Wang, X., Yuan, M., Zhang, X., Yang, X., Guan, X., et al. (2023). Identification of two novel T cell epitopes on the E2 protein of classical swine fever virus C-strain. *Vet Microbiol* 284, 109814. doi: 10.1016/j.vetmic.2023.109814
